# Recurrent pocket infection due to *Mycobacterium chelonae* at the site of an explanted cardiac implantable electrical device in proximity to a long-standing tattoo

**DOI:** 10.1016/j.hrcr.2015.10.012

**Published:** 2016-01-25

**Authors:** John Whitaker, William Rickaby, Alistair Robson, Farrah Bakr, Jonathan White, C. Aldo Rinaldi

**Affiliations:** * Department of Cardiology, St Thomas’ Hospital, London, United Kingdom; † Department of Dermatopathology, St John’s Institute of Dermatology, St Thomas’ Hospital, London, United Kingdom,; ‡ St John’s Institute of Dermatology, St Thomas’ Hospital, London, United Kingdom

**Keywords:** Cardiac implantable electrical device, Infection, Extraction, Mycobacterium

## Introduction

**Table:** KEY TEACHING POINTS

1. CIED infection is increasing because of the increased number of devices being implanted, the increased number of revision procedures, and the changing clinical profile of patients treated with CIED. 2. Successful treatment of CIED infection involves the combination of complete system removal and appropriate choice and duration of antibiotic therapy. The selection of appropriate antibiotic therapy depends critically on the identification of the causative organism. 3. Acid-fast bacilli (AFB) infection should be considered as a possible cause in cases of persistent superficial inflammation or discharge at surgical wound sites (including those following CIED extraction) despite empiric antibiotic therapy. The high prevalence of *Myocabcaterium chelonae* contamination of tattoo ink, which has been associated with the increased prevalence of tattoos, makes consideration of infection with this organism important. When AFB infection is suspected, specific samples should be collected for AFB culture as early as possible. In this case, diagnosis may have been reached earlier if samples had been taken for AFB culture when the device-extraction site was reexplored.

We present the first reported case of infection of a cardiac implantable electrical device with a nontuberculous mycobacterium. We also discuss the importance of conducting a thorough microbiological investigation to correctly identify the causative organism in cases in which initial investigations have negative results yet symptoms persist. In addition, the issues surrounding the increasing prevalence of *Mycobacterium chelonae* and its possible relationship to contaminated tattoo ink are discussed in the context of this case.

## Case report

**Figure 1:**
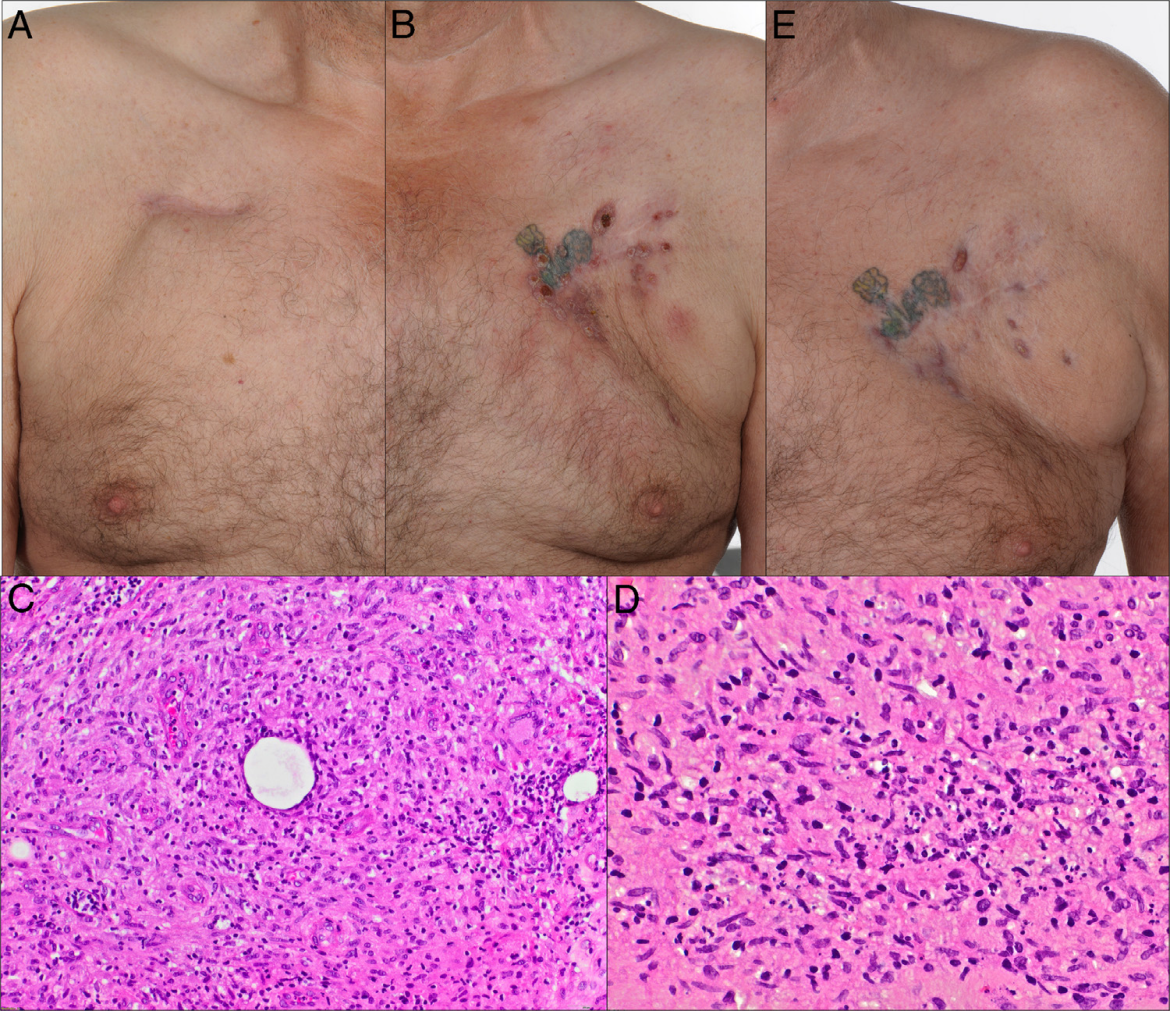
**A:** Right-sided CIED cardiac implantable electrical device (CIED) site. **B:** Chronically discharging and poorly healing wound at site of recent extraction of a CIED. **C and D:** Photomicrographs of skin biopsy with suppurative granulomatous inflammation and foci of necrosis. (H & E staining; D ×40, E ×100). **E:** Following prolonged treatment with doxycycline and clarithromycin, the discharge stopped and the irritation at the CIED extraction site settled.

A 69-year-old male with ischemic cardiomyopathy and a cardiac resynchronization therapy defibrillator (CRT-D) that had been implanted 6 years earlier was admitted with swelling and erythema around the site of his left-sided cardiac implantable electrical device (CIED) generator, which lay underneath a tattoo that had been present for 25 years. He was afebrile and clinically well, with no rigors, night sweats, or weight loss. Inflammatory markers on admission were mildly raised only—C-reactive protein concentration 7mg/L [normal range (NR) 0–4mg/L], white blood cell count 6.8 × 10^9^ cells/L (NR 4.0–11.0 × 10^9^ cells/L), neutrophil count 3.3 × 10^9^ cells/L (NR 1.5–7.0 × 10^9^ cells/L), and lymphocyte count 2.7 × 10^9^ cells/L (NR 1.2–3.5 × 10^9^ cells/L). Multiple samples were collected for blood culture, and no organisms were grown. A transthoracic echocardiogram demonstrated moderately to severely impaired left ventricular function. The right ventricular lead was thickened, but no vegetations were visualized. The patient underwent positron emission tomography with 6-fluoro-6-deoxy-D-glucose (6FDG) that demonstrated significant 6FDG uptake at the superior margin of the CRT-D generator and in a collection inferior to the generator. In addition, there was increased FDG uptake along the course of the right atrial pacing lead. The patient was treated with intravenous flucloxacillin and underwent CIED extraction under general anesthesia. Approximately 50 mL of pus was drained from a left infraclavicular incision over the site of the CRT-D generator; a sample of this drainage was sent for microscopy and culture. The generator was mobilized and the leads removed. The right atrial lead was withdrawn using gentle traction, a locking stylet was used for the right ventricular lead, and the left ventricular lead required a laser sheath for removal from the coronary sinus. The lead tips and an excised portion of infected tissue were sent for microscopy and culture. After evacuation of pus and removal of the implanted material, the wound was treated with hydrogen peroxide and closed using Vicryl sutures, as is our standard approach after full debridement. The patient was in sinus rhythm with intact atrioventricular conduction and a satisfactory underlying heart rate, so he did not require a temporary device. He had a further week of treatment with intravenous antibiotics. A week after the device was extracted, he underwent reimplantation with a right-sided CRT-D (Figure 1A) and was subsequently discharged clinically well. The fluid drained from the generator pocket was purulent. Pus cells but no organisms were seen on microscopy, and there was no growth on prolonged culture.

Following his discharge from the hospital, the patient noted poor skin healing around the site of the CIED extraction (Figure 1B). He reported discharge from the left infraclavicular region, at the site of his previous CRT-D device, and the area was intermittently itchy and tender. Multiple samples of the discharging fluid and skin swabs were taken during this period and cultured. One sample showed growth of *Staphylococcus epidermidis* (coagulase-negative staphylococcus) only. The patient was treated empirically with 2 courses of oral antibiotics, 1 week long each, and 1 course of intravenous antibiotics, 2 weeks long, and his symptoms, which improved during the administration of the antibiotics, returned with completion of each antibiotic course. Four months following the device extraction, he underwent reexploration of the pocket, and further samples were collected for culture. No retained material was identified at the time of reexploration. Small numbers of pus cells only were seen on microscopy of the tissue samples taken at reexploration. No organisms were seen on initial microscopy or following prolonged culture. Acid-fast bacilli (AFB) cultures were not collected at reexploration.

Because of ongoing symptoms of discharge from around the poorly healed left-sided site of the extracted device, the patient underwent a skin-punch biopsy. The skin-punch biopsy was taken from erythematous skin overlying the site of the extracted device. It was adjacent to, but did not include, skin stained with tattoo ink. The skin-punch biopsy demonstrated marked suppurative granulomatous inflammation (Figures 1C and 1D). Histochemical results, including Ziehl–Neelsen staining, failed to demonstrate organisms. No AFB were seen on the initial smear from the punch biopsy; however, following prolonged AFB culture, *M chelonae* that was sensitive to doxycycline and clarithromycin was found.

The patient underwent 6 months’ treatment with both doxycycline and clarithromycin. After 4 weeks off treatment, the discharge from the previous CIED site stopped, and there were no further episodes of tenderness or swelling (Figure 1E). There has been no recurrence of his symptoms 3 months following completion of the course of doxycycline and clarithromycin.

## Discussion

*M chelonae* is a group of organisms classified as nontuberculous mycobacteria (NTM). This group includes mycobacteria species other than *Mycobacteria tuberculosis* and *Mycobacteria leprae.*^1^ NTM are further classified according to their rate of growth. The pathogen in this case produces mature growth on media plated within 7 days and is therefore classified as a rapid-growing mycobacterium.

The precise incidence of *M chelonae* (and other NTM) skin and soft tissue infections is unknown^1^ but appears to be increasing.^2^, ^3^ *M chelonae* can be isolated from environmental, nonhuman animal, or human sources.^1^ It is thought that mains water supply is a major reservoir of NTM,^4^ and they have been isolated from up to 90% of mains water samples where they have been sought.^5^, ^6^ They produce an effective biofilm that can result in resistance to common disinfectants, including chlorine.^7^, ^8^ Infection may result in disseminated cutaneous disease (most commonly in the context of systemic immunosuppression), localized cellulitis, abscesses, or osteomyelitis.^1^ Infections around surgical wounds may present as wound infections, fistulae, or tracts that drain or feature skin nodules.^9^ They have also been reported as chronic, nonhealing skin ulcers, similar to that which occurred in our patient.

Of particular interest in this case is the association between *M chelonae* infection and tattoo ink. A previous public health investigation identified tattoo ink as the source in outbreaks of *M chelonae* skin and soft tissue infection in Scotland.^10^ In this report and systematic review, the focus was on recently tattooed skin (within the previous year) that subsequently developed NTM skin or soft tissue infection. In such outbreaks, it is commonly postulated that the infection is the result of contaminated tattoo ink and in certain cases it is thought to be the result of diluting tattoo ink with water from the mains supply.^10^ Recent investigations have demonstrated high rates of bacterial contamination (although investigators were not specifically looking for NTM) of tattoo ink. In unopened bottles, the rates were as high as 10%, and in addition there were high rates of inadequate sealing of bottles once they were opened.^10^ Given the gap between the tattoo ink introduction and the infection in the current case, it is uncertain whether the infection represents reactivation of NTM that was introduced by the original tattoo ink or whether it represents de novo exposure and infection. No guidelines are currently available for the treatment of skin and soft tissue infections with *M chelonae,* but expert advice is that monotherapy should be avoided and prolonged treatment is usually required.^1^

## Conclusion

The current case is of interest as it is the only reported case in the literature of CIED pocket infection due to *M chelonae*. The recurrence of inflammation despite full system removal and the lack of positive microbiologic results are notable points and should raise the suspicion of atypical causes of infection, such as mycobacterium. The incidence of CIED infection^11^ and the incidence of NTM infection are rising. An awareness of NTM as possible causative organisms in superficial infections associated with CIED is important because of the particular conditions required to successfully isolate these organisms and the prolonged treatment necessary to eradicate them if they are identified as causative organisms.
